# JUNGFRAU detector for brighter x-ray sources: Solutions for IT and data science challenges in macromolecular crystallography

**DOI:** 10.1063/1.5143480

**Published:** 2020-02-26

**Authors:** Filip Leonarski, Aldo Mozzanica, Martin Brückner, Carlos Lopez-Cuenca, Sophie Redford, Leonardo Sala, Andrej Babic, Heinrich Billich, Oliver Bunk, Bernd Schmitt, Meitian Wang

**Affiliations:** Paul Scherrer Institut, Forschungsstrasse 111, 5232 Villigen PSI, Switzerland

## Abstract

In this paper, we present a data workflow developed to operate the adJUstiNg Gain detector FoR the Aramis User station (JUNGFRAU) adaptive gain charge integrating pixel-array detectors at macromolecular crystallography beamlines. We summarize current achievements for operating at 9 GB/s data-rate a JUNGFRAU with 4 Mpixel at 1.1 kHz frame-rate and preparations to operate at 46 GB/s data-rate a JUNGFRAU with 10 Mpixel at 2.2 kHz in the future. In this context, we highlight the challenges for computer architecture and how these challenges can be addressed with innovative hardware including IBM POWER9 servers and field-programmable gate arrays. We discuss also data science challenges, showing the effect of rounding and lossy compression schemes on the MX JUNGFRAU detector images.

## INTRODUCTION

Macromolecular crystallography (MX) is the dominant method for high-resolution structure determination of biomolecules. Currently, the protein data bank (PDB) has about 140 000 x-ray structures and most of them were determined at synchrotron facilities. Besides, numerous protein-ligand structures are determined routinely for structure-based drug discovery in the pharmaceutical industry. Along with the continuous development in beamline instrumentation, particularly hybrid pixel-array detectors (PAD), diffraction-data collection methods have evolved as well. The traditional high-dose, low multiplicity, and coarse slicing data collection strategy^1^ has been gradually replaced at third-generation synchrotrons by continuous shutterless data collection with low-dose, high multiplicity, and fine slicing methods^2–5^ thanks to the fast frame-rate, high sensitivity, and low-noise of PAD.^6^ The PAD also enabled fast x-ray based scanning (i.e., sample rastering) for detecting weakly diffracting microcrystals. From each identified crystal, a partial data set is then collected and many of them are assembled in a method called serial synchrotron crystallography (SSX).^7^ In addition to the rotation method at cryogenic temperature, room-temperature (RT) MX with still diffraction images has emerged for studying protein dynamics in recent years. This RT serial crystallography has enabled time-resolved crystallography to reach ps to ms resolution at x-ray free-electron laser (XFEL)^8,9^ and synchrotron^10,11^ facilities, respectively.

In parallel to the rapid advances of single-photon counting technology,^12^ next-generation charge-integrating detectors are maturing. One of such detectors is the adJUstiNg Gain detector FoR the Aramis User station (JUNGFRAU),^13–15^ which features direct detection, a high dynamic range, a linear photon-rate response, and a superb spatial response. JUNGFRAU has low electronic noise: for a short integration time (10 *μ*s), it is 83 e^−^ RMS and for a long integration time (840 *μ*s), the value increases to 200 e^−^ RMS.^13,16^ In both cases, when data are converted to photon counts, this noise is a small fraction of a charge generated by a single x-ray photon, e.g., 3500 e^−^ by a 12.4 keV photon. JUNGFRAU can be operated with a frame rate of up to 2.2 kHz. With the next-generation synchrotron sources [i.e., Diffraction Limited Storage Rings (DLSRs)] on the horizon, higher photon flux and tighter beam focus will be available at MX beamlines.^17^ With such source brilliance, rotation crystallography could be carried out in a second at an unprecedented speed of 100°/s or higher, experimental phasing could be conducted at lower energy than what's possible today, and serial crystallography could be performed as continuous scans in kHz frame rate for both static and time-resolved crystallography.^18^ At XFEL sources operated at a high repetition-rate (up to MHz), a fast frame-rate integrating detector can significantly improve data collection efficiency and reduce sample consumption. These scientific opportunities are exciting and promising. The JUNGFRAU detector could potentially play a transformative role; however, the operation of a large-format detector at kHz frame rates comes with its own technical challenges. If such challenges are not addressed in a technically sound and economically sustainable manner, the scientific impact of the next-generation light sources could be compromised. Here, we present our solutions to operate the JUNGFRAU detector at synchrotron protein crystallography beamlines from high-performance computing and data science points of view. We will explain how JUNGFRAU specifics affect data representation, we will compare possible compression schemes with their consequences and we will show hardware and software solutions necessary for the 46 GB/s data-rate from a JUNGFRAU 10 Mpixel detector planned for Swiss Light Source macromolecular crystallography (MX) beamlines.

## OVERALL DATA FLOW

**FIG. 1.**
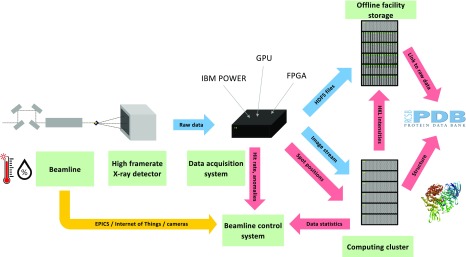
Data flow envisioned for kilohertz framerate JUNGFRAU detectors at the Swiss Light Source MX beamlines. Blue arrows represent the flow of x-ray images (the most throughput critical), red arrows the flow of metadata, and the yellow arrow the flow of sensor information.

The schematics of data flow envisioned for JUNGFRAU at Swiss Light Source MX beamlines are presented in Fig. 1. After each image is recorded on the detector, the pixel content is transferred via a fiber optic Ethernet network to the data acquisition system. The system needs to perform the following steps:

1. All the incoming packets are processed and sorted losslessly.
2. Gain and ADU (arbitrary detector units) pixel content is converted into energy or photon count units, accounting for the special larger pixels between detector chips—i.e., splitting these into two or four pixels in the output image, so all pixels are of the same size. In this step, multiple frames can be summed to reduce the frame rate as well.
3. The full image is composed and analyzed for features like the Bragg spot number and positions, ice rings, and salt crystal reflections. Optionally, a veto mechanism can be used to discard useless frames. There is also the option to inform the beamline operator on the presence of anomalies that could compromise the experiment and detector (e.g., empty frames, suspiciously strong reflections).
4. The composed image is compressed and written into external storage.
5. Further analysis with MX software is performed with online and offline processing systems.

This publication covers steps 1–4, aiming to optimally implement them in a single computing system.

## DATA RATES

The JUNGFRAU detector, similarly to EIGER, is a modular detector. A single JUNGFRAU module, a basic building block, has 524 288 pixels organized into 1024 columns and 512 rows. Each module has an independent readout with two 10 Gbit/s Ethernet (10 GbE) ports per module.

Since one pixel is encoded by 16 bits, the network interface is limiting the frame rate. In the case of a single 10 GbE interface, 1.1 kHz is achievable, with a data rate of 524 288 pixels × 16 bit × 1.1 kHz = 1.15 GB/s. This is 9.23 Gbit/s and is very close to theoretical bandwidth of 10 GbE, especially as the calculation does not account for overheads from the network protocol and detector headers. Enabling a second 10 GbE interface doubles the maximal frame rate of a single module to 2.2 kHz and data rate to 2.30 GB/s.

Data rates for larger system JUNGFRAU detectors are obtained just by multiplying the numbers presented above by the number of modules. The summary of operated and planned systems for macromolecular crystallography at the Paul Scherrer Institut is presented in Table I. For reference, these data rates can be compared with the first large format PAD installed at the Swiss Light Source X06SA beamline in 2007, i.e., PILATUS 6M. The detector, state-of-the-art at the time, operated with a 12.5 Hz frame rate and produced roughly 300 MB/s data without compression.^19,20^ Another reference point is the fastest commercially available large format x-ray detector for MX applications, which at the time of writing is Dectris EIGER2 XE 16M, producing 13.4 GB/s at a steady 400 Hz frame rate or bursts of 18.5 GB/s at a higher 550 Hz frame rate.^21^

**TABLE I.** Summary of data rates in GB/s for large format JUNGFRAU detectors used for macromolecular crystallography at the Paul Scherrer Institute.

Application	Detector size (Mpixel)	Number of modules	Frame rate (kHz)	Data rate (GB/s)
SwissFEL	16	32	0.1	3.4
Swiss Light Source (2018)	4	8	1.1	9.2
Swiss Light Source (2021)	10	20	2.2	46.1

## DATA RECEIVING

To achieve the highest possible utilization of the network interface and real-time operation, a simple transport layer protocol, User Datagram Protocol (UDP), needs to be used for sending data from the detector to a readout computer. In such a protocol, network infrastructure does not guarantee delivery of a packet from the sender to receiver. If for example receiver buffer is flooded, incoming packets will be dropped and never recovered. However, it is practically a better strategy, as protocols with a mechanism for reliable packet delivery, like widely used Transmission Control Protocol (TCP), introduce overhead on communication and require buffering capabilities.

Since receiving data at rates of multiple GB/s is a challenging task, it requires using high-end servers with multiple CPUs installed and a large amount of memory for buffering. We used a system with 4 Intel Xeon CPUs and 1.5 TB random access memory (RAM) installed (see Methods). Such systems have a non-uniform memory access (NUMA) architecture. While placed in a single box, the system is built from 4 NUMA nodes, each having a single central processing unit (CPU), a quarter of system memory, and Peripheral Component Interconnect Express (PCIe) extension cards. Each of the NUMA nodes has dedicated fast interconnect to all three others. However, accessing memory or devices by a program running inside a single node is faster, than if communication is scattered over multiple nodes. For convenience, these features are usually hidden from the user, who programs the machine as a single box.

However, we found that eliminating cross-traffic across NUMA nodes was indeed necessary for handling the JUNGFRAU 4 Mpixel detector at 1.1 kHz. To achieve this, we mounted 4 Mellanox Connect-X 4 Lx network cards in the machine, each having two ports for 10 GbE, in a way that each card was connected to a different CPU/NUMA node. Each card was servicing 2 JUNGFRAU modules. We ran one receiver process per module. Each process was pinned to a CPU that belonged to the same NUMA node as the network card. Similarly interrupts of the network card were pinned to a respective CPU, and different CPU cores were used for receiving and interrupt handling. Received data were saved to a RAM disk using only memory inside the particular NUMA node.

In this experiment, we aimed to save the raw data in memory and do further steps of the data flow after the collection is finished. Indeed, we could successfully fill memory with the data - allowing us to save 2 min 20 s of continuous exposure at 1.1 kHz. We also found that using four 3.2 TB fast SSD disks, mounted as PCIe 3.0x4 cards, in a similarly NUMA aware configuration, allows us to store about 20 min of exposure without lost frames.

Receiving the data with standard methods is limited by the network stack implementation in Linux. For the secure and versatile operation of multiple network applications on a single system, the Linux kernel needs to analyze and sort all the incoming packets. This requires a context switch between user application and kernel, as well as several memory copies between internal buffers before data are received by the proper application. The benefit of using the operating system network stack is portability—receiver can work on any hardware that supports Linux kernel. However, when performance is the key factor, and the receiver is expected to react to only one type of traffic—UDP/IP packets sent by the detector, a more optimized solution can be selected.

Network cards have an ability for user-space Ethernet, also called raw Ethernet or zero-copy transfer. This instructs the network card to directly write incoming network packets into a user-space buffer, avoiding kernel involvement. It is the user application that decodes the content of the incoming packets, including any network or transport layer protocol headers.

We have tested the functionality as implemented in the *ibverbs* library for the Mellanox Connect-X network cards.^22^ Although the library implements the remote direct memory access (RDMA) protocol, we have chosen the option to set the queue pair as IBV_QPT_RAW_ETH, thus treating any incoming Ethernet traffic as if it were an RDMA communication. This is different from data acquisition frameworks that implement RDMA for both the receiver and sender.^23^ In the case of raw Ethernet, the sender of the data (the detector) is not aware of RDMA, and it sends standard UDP/IP packets. It is only the receiver that uses the RDMA API.

As a conventional approach, with the Linux kernel stack, is not sustainable for increasing data rates (>20 GB/s), we have tried the Mellanox raw Ethernet functionality, as a proof of concept, for future use with 2.2 kHz 4M and 10M JUNGFRAU. For the test, we have taken a JUNGFRAU 4 Mpixel detector operating at 1.1 kHz. In this case, all traffic from all eight modules of the detector was routed to a single 100 GbE Mellanox card, spread over two 40 GbE ports due to a switch limitation. Two parallel threads were used, each receiving data from a single 40 GbE port. After a frame was received, pixel readout was copied from the packet to a second array. We have tried collecting up to 20 000 frames and all were successfully received without lost frames, and the size was limited by the memory capacity of a smaller 2-socket Intel Xeon server used for the test. The user-space technology is, therefore, a promising solution to implement for high data rate detectors.

An even more performing solution is replacing the network card with a field-programmable gate array (FPGA) mounted on a PCIe board to process the incoming detector traffic. By proper design, FPGA can analyze arriving packets, select ones that were sent by the detector, decode the frame header, and put the packet content into designated space in memory. As FPGA functionality is encoded in hardware, latency and throughput are predictable, so this is an attractive solution for a real-time processing system. However, the effort of developing FPGA implementation is higher than CPU programming.

## JUNGFRAU PIXEL ENCODING

JUNGFRAU is a gain adaptive detector. During exposure, each pixel can operate in three amplification modes (gain levels), designated as G0, G1, and G2. G0 is a base mode, which is sensitive to weak signals - allowing single-photon sensitivity. However, if integrated charge is close to G0 saturation, the detector will automatically switch first to G1 and later G2. These two modes allow measuring a stronger signal, and hence extend the total dynamic range. The choice of breakpoints between the three gain levels ensures that detector inaccuracies are below Poisson statistical uncertainty. The readout value from a JUNGFRAU pixel has to contain information on both the gain level and the stored charge (in ADU). As JUNGFRAU has a 16-bit pixel readout, the first 2 bits encode the gain level and the remaining 14 bits the accumulated charge.

To calculate the actual energy that was deposited in the pixel during the exposure, three tasks need to be done, as presented previously:^15^

1. The gain level needs to be decoded by interpreting the first two bits (00–G0, 01–G1, 11–G2).
2. From the charge value of 14-bit, one needs to subtract a “pedestal”—the mean value of dark noise, i.e., the mean reading of each pixel in the absence of x-rays. The pedestal is pixel specific and is different for the three gain levels, i.e., chosen accordingly. Pedestal values depend on system temperature and are affected by potential radiation damage of the sensor, so they need to be measured for the particular detector setup. In our case, a pedestal for low gains (G1 and G2) is collected in a dedicated run, as the detector needs to be forced to operate with these gains. To calculate the pedestal value for the highest gain (G0), the acquisition is started a few seconds before the shutter opening, and dark frames at the beginning of exposure are used.
3. Results of (2) need to be multiplied by a conversion factor (gain factor) from ADU to either energy expressed in eV or photon counts. Again, it is a pixel specific factor and different for each of three gain levels. Gain calibration is not affected by the particular detector setup and is done before detector operation, details were described by us beforehand.^15^

The procedure outlined in points (1)–(3) is sufficient for XFELs and pulsed measurements at synchrotrons, where a short integration time (e.g., 10 *μ*s) is used and the pedestal remains stable.^14^ Conventional operations at synchrotron MX beamlines are more difficult due to a relatively long integration time required to achieve the full duty cycle (∼450 *μ*s at 2.2 kHz). In this case, the detector system can take a few seconds to reach a stable operation state at the start of the measurement. During this time, there are small temperature changes in the whole system, which results in the drift of pedestal. The drift was observed at the level of up to 100 ADU (2.5 keV), which is a fraction of a photon. Currently, the effect is mitigated by the procedure, which introduces a delay between the starting detector and experiment, i.e., measuring photons and by pedestal correction. The pedestals are also monitored and updated throughout the measurement to take the drift into account.^14^ This comes at the expense of a slightly increased computational complexity and time. Alternatively, the detector could be operated in a continuous mode, where it is always measuring, but only relevant frames, determined by a beamline trigger signal, are saved.

Another correction, common mode, is often used for integrating detectors.^24^ It accounts for spatially correlated fluctuations of the pedestal. Although such noise is also present in JUNGFRAU, the low magnitude of the fluctuations makes the correction unnecessary for MX applications. However, other applications where pixels are summed together, e.g., spectroscopy, might benefit from including it.

## JUNGFRAU IMAGE CONVERSION

The raw data from the JUNGFRAU detector cannot be directly processed by standard macromolecular crystallography software. There is a need for a conversion from a 16-bit gain+ADU representation to a linear scale of either energy deposited in the pixel or photon count. While the procedure is very straightforward, the difficulty in executing it comes from the huge amount of data needed for processing.

**FIG. 2.**
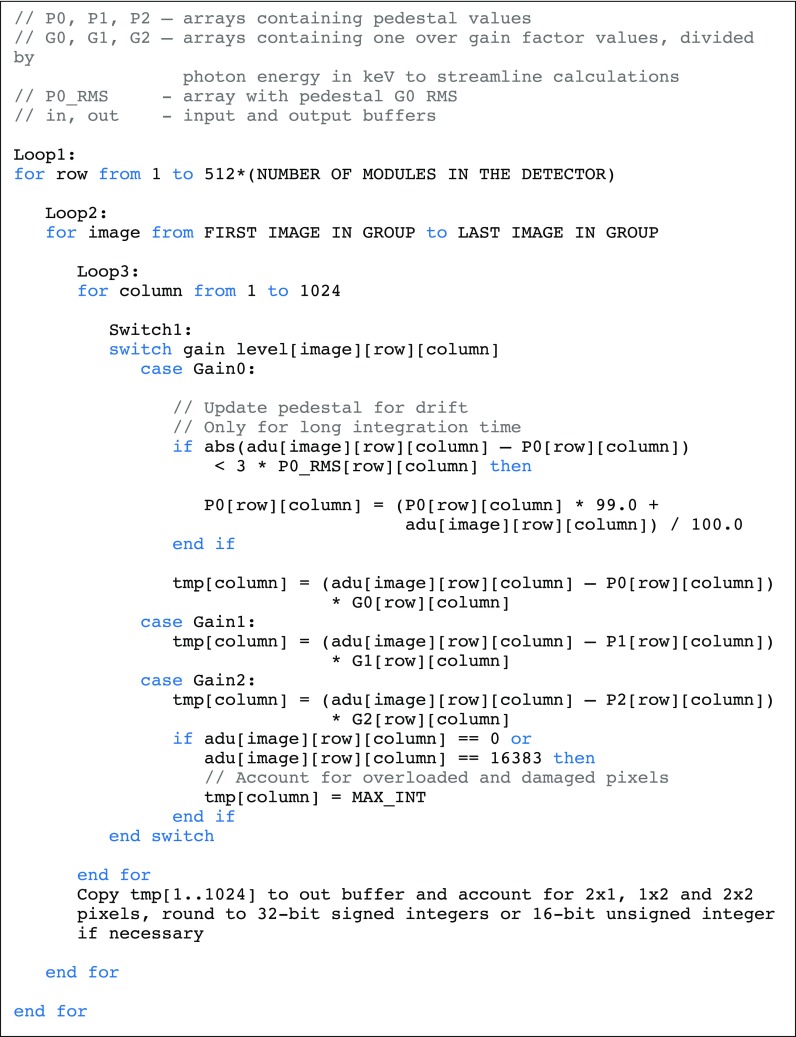
Pseudo-code for the JUNGFRAU data conversion procedure without frame summation.

Pseudocode, implementing the conversion procedure explained previously,^14,15^ is presented in Fig. 2. The code requires executing 2 floating-point operations per single pixel—one subtraction to account for the pedestal and one multiplication/division to account for signal amplification at a given gain level. Calculating for a 4 Mpixel detector, operating at 1.1 kHz frame rate, one needs to process approximately 5 × 10^9^ pixels per second. Multiplying by 2 floating-point operations (FLOP), this means 10 × 10^9^ floating-point operations per second (GFLOPS), which is extremely small for current computing systems. For example, for a single Nvidia V100 general-purpose graphics processing unit (GPGPU), the producer claims 7 TFLOPS peak performance,^25^ more than two orders of magnitude, so the task is not limited by the calculating capacity of modern computing systems. However, there is another factor—memory, which is more limiting in that case.

To convert a single pixel, CPU needs to fetch six constants from memory: pedestal value and gain factor for all three gains. Since these values are 32-bit single-precision floats, the combination of six values requires fetching 192 bits per pixel. For the 5 × 10^9^ pixels per second mentioned above multiplied by 224 bit (16 bit = pixel in + 16 bit = pixel out + 192 bit = conversion constants), one needs to be able to achieve steady data transfer from RAM of 129 GB/s. The situation is more daunting for the 10 Mpixel 2.2 kHz detector, where the number is 5× larger, 646 GB/s. Accounting for pedestal drift increases memory needs, as one extra constant (the pedestal G0 RMS) has to be fetched, and the updated G0 pedestal has to be written to memory, resulting in higher rates of 166 GB/s for 4 Mpixel 1.1 kHz and 830 GB/s for 10 Mpixel 2.2 kHz. To give the proper magnitude—the HPE DL580 Gen10 server used in our test measures 310 GB/s memory bandwidth in STREAM test (copy), when using all CPUs.^26^

**FIG. 3.**
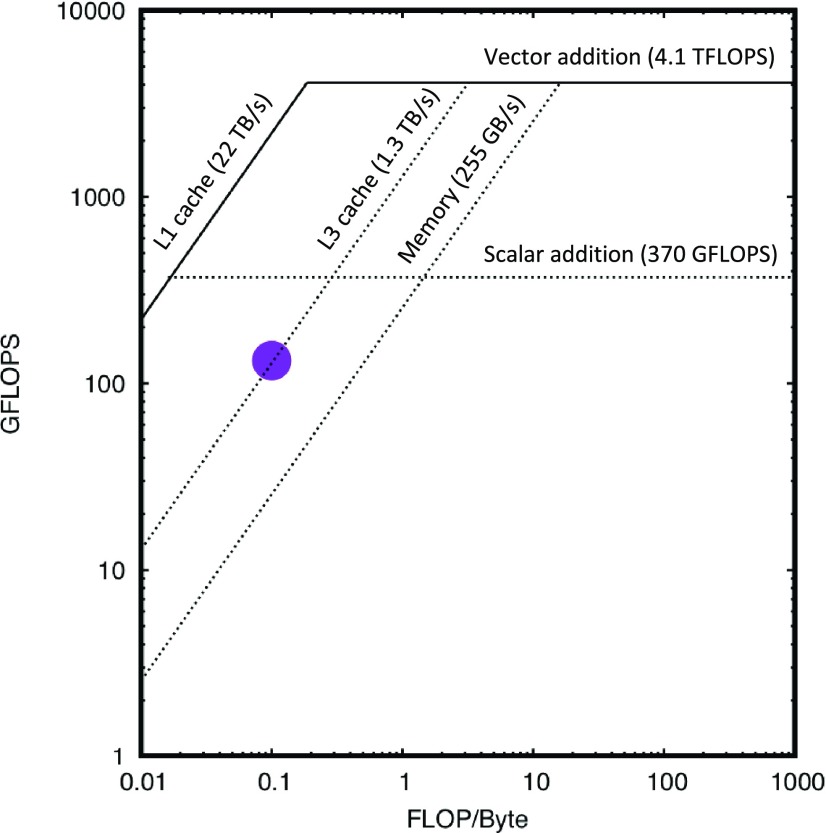
Roofline analysis is a method to compare the performance of a current implementation (loop, function) with the best possible for given hardware. Two values are taken into account—arithmetic intensity, i.e., the number of floating-point operations per volume of data (X-axis) and performance, i.e., the number of floating-point operations per unit of time (Y-axis). Dotted lines represent “ceilings”—horizontal lines correspond to limits on the number of CPU operations, while diagonal lines represent bandwidth limitation of memory and CPU cache. A purple dot represents the performance of Loop3 on Fig. 2 (no frame summation)—since the dot is positioned above the DDR memory ceiling, it shows that the procedure is using the full performance of CPU cache of level 3 (L3). Both loop performance and roofline limits are measured with Intel Advisor 2019 and are aggregated over 48 cores. The number of floating-point operations per second is calculated over loop execution time only.

To achieve the highest conversion performance, the code was adapted to benefit from a single instruction multiple data (SIMD) model, present in modern CPUs and GPGPUs. As an example of SIMD, Advanced Vector Extensions 512 (AVX-512) capable Intel Xeon CPUs have 32 512-bit registers (called also ZMM registers). Each such register can store at once 16 32-bit single-precision floating-point numbers or 8 64-bit double-precision floating-point numbers. Next, the CPU can perform the same operation simultaneously on each of the numbers stored in the wide register, e.g., addition or multiplication. This is fully equivalent to performing 16 scalar additions but requires only one instruction instead of 16. The SIMD code can be automatically generated for loops with modern compilers (see Methods). Used of SIMD increases the performance roofline significantly (see Fig. 3), but comes at a cost for loops having conditional branches—in this case, the CPU will execute all branches and will select the correct result when data are written to memory. For the Switch1 statement (Fig. 2), the CPU will calculate the photon count value for all three gain levels, but will only write the correct one to memory. This means reading from memory all the conversion constants, irrespective of whether, for example, only the G0 gain level was present in the data. As indeed most pixels never switch gain in MX measurement, function could first check, if a chunk of the data contains pixels that switched to lower gain levels (G1 and G2)—and if these are absent, for that particular chunk to execute code that implements only the G0 branch of Switch1 statement (Fig. 2). This would reduce memory throughput needs, as fewer constants are transferred. With the current implementation, such optimization did not improve performance, but it could be helpful in the future. A drawback of such optimization is that performance would be less predictable, as it would depend on the image content.

The code was parallelized with the POSIX threads (pthreads) library, allowing for fine-grained control of access to variables with mutexes and monitors. Two categories of threads were implemented—conversion threads that implement the arithmetically intensive part of the task and file writer threads that also cover data compression. The separation is important, as for optimal operation each converter thread needs to cover a small area of the detector for all images, while converter threads need to have images composed of all modules, but can operate on only a subset of frames. Loop ordering affects memory bandwidth. If a single vertical line is converted for a subset of frames, e.g., 100, conversion constants for this line can be kept in the CPU cache and the number of memory operations is significantly reduced.

During the JUNGFRAU 4M commissioning at the Swiss Light Source, we tested the code for conversion of JUNGFRAU images to photon counts. For a 4 Mpixel detector, running at 1.1 kHz, the time to perform all the conversion steps is approximately twice the data collection time. As a test case, we chose a lysozyme crystal, rotated for 720° at 100°/s, recording 0.088° per image. The total collection time was 9.2 s without the beam to allow for pedestal drift to stabilize and 7.2 s with beam crystal rotation.

The overall time to run the full conversion procedure on these data—i.e., to analyze pedestal images, generate pedestal maps, convert protein diffraction images to photon count units, track pedestal modifications, and save results as a Hierarchical Data Format version 5 (HDF5)^27^ file was 14 s when running in isolation on the server mentioned above. However, if we allowed for coarser slicing of 0.44°/image, with one output image being the summation of five detector frames, the conversion time reduced, to 9.2 s, equivalent to data collection time. As summation is the last step of conversion, the number of instructions for conversion remains the same; however since fewer frames are transferred to the writer process, the program spends less time on compression and output, improving the overall performance. With roofline plot analysis (see Fig. 3),^28^ we show that the conversion procedure is close to architectural limits of CPU cache performance. So, while the CPU procedure could be used for online conversion for small size detectors (1 Mpixel) or at frame rates below 100 Hz, it is an order of magnitude too slow to be a sustainable solution for large format detectors operating at 2 kHz frame rate.

Alternatively, the conversion routine can be implemented in FPGA, with guaranteed latency and throughput. As PCIe FPGA boards are equipped with 100 GbE ports, it is possible to combine the receiving and conversion function in a single FPGA—here only converted data are written to host memory and the number of memory copies is reduced. Both properties make the solution especially attractive for a real-time processing system and are less important for offline analysis. As a proof of concept, we have developed the C code for the routine that can be compiled into register transfer language for FPGA via Xilinx High-Level Synthesis.^29^ The main modification is that the floating-point arithmetic used on the CPU is replaced with fixed-point representation. We have tested our implementation, where the pedestal for G0 is implemented as a 22-bit fixed-point number keeping extra precision for drift update, while all the other constants are 16-bit fixed-point numbers. The measured RMS on a small dataset between CPU floating-point implementation to single 12.4 keV photons and FPGA fixed integer, also rounded to single photons, is 0.22 photon, which is a reasonable value for rounding to full photons. According to the FPGA synthesis result, this design is capable of converting 32 pixels per single clock cycle at 250 MHz frequency allowing for 16 GB/s conversion speed, more than 12 GB/s that 100 GbE can provide. As the resource utilization is around 10% of the XCVU33P FPGA logic elements (see the Methods section for details regarding the chosen FPGA), there is a possibility to duplicate the design to process 32 GB/s within a single FPGA board (2 × 100 GbE) or to add more functionality, like online spot finding or machine learning (ML) inference. With such design in operation, two FPGA boards would suffice to handle JUNGFRAU 10M at the full frame rate.

A middle ground between CPU and FPGA would be a GPGPU implementation with more SIMD cores than CPU and better memory bandwidth. Software development and optimization are simpler for GPGPU than for FPGA. While GPGPUs are not real-time devices, it is anyway easier to control execution time, as only one kernel is executed on a GPGPU. A GPGPU version was also developed with promising performance results, which will be explained in another publication.

## PIXEL REPRESENTATION AND COMPRESSION

The lowest number a pixel can encode per exposure is 1 ADU at G0, corresponding to roughly 0.005 photons at 12.4 keV. The highest number, saturation at G2, is roughly 10 000 12.4 keV photons. The ratio between the two is 2 × 10^6^, so one needs a 21-bit integer to encode all possible outcomes of the JUNGFRAU pixel on a linear scale. As CPUs operate with numbers of only a given bit width, 32-bit fixed-point representation is a natural choice, as the range of pixel photon counts is fixed, although 32-bit floating-point representation could also be used for calculation convenience.

The highest possible precision is necessary when energy or sub-pixel position of photons is measured. This is the case when fluorescence photons need to be distinguished from ones having incoming beam energy.^30^ However, the full 21-bit precision is not always necessary—e.g., in the case of protein diffraction with a monochromatic beam, the most relevant total is the integrated number of photons per reflection.

To test the effect of rounding, we applied various schemes to a lysozyme dataset collected with the JUNGFRAU using the rotation method. Table II summarizes the results with different rounding schemes. While rounding coarser than one photon results in a clear reduction of both data quality in high resolution shells, refinement statistics, and total anomalous signal, the effect of rounding finer than one photon is very small for precision indicators (R_meas_ for the highest resolution shell) and not visible for accuracy measures (R_free_, mean anomalous peak height). This is expected, as rounding coarser than one might “lose” photons after integrating. However, rounding to exactly one photon will lose a photon only in a very specific condition, when the reflection observation contains only one photon and this photon hits a four-pixel junction. This is similar to the “corner effect” explained by us previously for photon counting detectors,^13^ although applying only to the weakest, single photon, observations. Measurement error for these reflections is dominated by random noise, coming from crystal quality and counting statistics.^31^ As, however, the rounding error does not scale up with the intensity of the reflection, it will be negligible for strong reflections, the ones that are most sensitive for the systematic error of the detector. This is different from the corner effect observed for photon counting detectors, where photon loss is increasing with more photons for a Bragg spot, leading to systematic error for low resolution. These considerations are also consistent with our prior work, where we used rounding up to full photons and we observed that the positioning of Bragg spots in relation to the sub-pixel position does not affect spot intensity.^13^

**TABLE II.** Data quality indicators in function of rounding the JUNGFRAU pixel readout value to a multiple of photon count for the lysozyme crystal dataset collected at the Swiss Light Source X06SA beamline with the JUNGFRAU 4M at 1.1 kHz using 12.4 keV x-rays, 100% beam transmission, and 0.088°/880 *μ*s steps. 2045 images (180° rotation) were taken for data analysis. The low resolution shell is defined as 50–3.25 Å, while the high resolution shell is 1.18–1.31 Å. The size of the dataset was calculated after compressing with Bitshuffle/LZ4.

Rounding to a multiple (photons)	R_meas_ low/high res. shell (%)	Mean anomalous peak height for S (σ)	Refinement statistics R_work_/R_free_ (%)	Bitshuffle/LZ4 compression (bits/pixel)
1/8	2.2/18.2	15.3	11.7/13.6	5.0
1/4	2.2/18.3	15.1	11.7/13.4	4.1
1/2	2.1/18.5	15.2	11.6/13.4	3.1
1	2.1/18.9	15.2	11.6/13.5	2.3
2	2.2/22.8	14.7	11.8/13.5	1.5
4	2.1/27.8	14.3	12.5/14.5	0.90
8	2.1/30.6	13.9	15.4/17.6	0.39

The next step was to analyze different compression schemes. A similar study was done in the past when the EIGER detector was introduced.^32^ It was established at the time that the best compression factor was obtained using a two-step method. The algorithm consists of two steps. In the first step, the Bitshuffle filter,^33^ positions of bits are exchanged. It takes 16 consecutive 16-bit integers (or 32 × 32-bit integer) and reorders bits, writing highest bits for all numbers first, then second-highest, third-highest, etc. As most diffraction images are composed of relatively homogeneous background, low counts of similar magnitude, most blocks will be made of a long sequence of zeros with few ones close to the end, making it easier for a dictionary compression, e.g., Liv-Zimpel 4 (LZ4),^34^ as predictable sequences will be longer. LZ4 was chosen as it is optimized for decompression performance. As compression is an active field, new algorithms are introduced, we have also included in our tests new algorithm from LZ4 author called Zstandard (Zstd).^35^ The algorithm is similar to LZ4, as made for fast decompression, but is expected to offer better compression factors. Finally, we also tested Gzip, as it is the most commonly available algorithm at the moment.

The results of compression tests are presented in Table IV for the total conversion process and Table S1 for pure compression. Indeed, a combination of Bitshuffle and a compression algorithm gives the best results, with Bitshuffle/LZ4 offering the highest throughput and Bitshuffle/Zstd offering the highest compression ratio. As the Bitshuffle compression scheme is a two-step process, it is usually performed in blocks fitting CPU cache, usually 8 kB, Table S2 presents the relationship between the block size and compression, showing that Bitshuffle/Zstd needs longer blocks to perform better, while Bitshuffle/LZ4 does not benefit from increasing block size and a block size of 64 kB is recommended for Bitshuffle/Zstd. Gzip is an order of magnitude slower, without any gain in the compression ratio. Interestingly, as summarized in Table IV, we have found that XDS data processing is not limited at the moment with decompression performance, with difference below 3% from the fastest to slowest and no reasonable trend.

**TABLE III.** Data quality indicators with SZ lossy compression for the lysozyme crystal dataset collected at the Swiss Light Source X06SA beamline with the JUNGFRAU 4M at 1.1 kHz using 12.4 keV x-rays, 100% beam transmission, and 0.088°/880 *c*s steps. 2045 images (180° rotation) were taken for data analysis. The low resolution shell is defined as 50–3.31 Å, while the high resolution shell is 1.18–1.11 Å. The number of reflection observations accepted per resolution shell is taken from the output of the XDS CORRECT step.

Absolute error bound in SZ	R_meas_ low/high res. shell (%)	Number of accepted observations; low/high res. shell	Mean anomalous peak height for S (σ)	Refinement statistics R_work_/R_free_ (%)	Compression factor (bits/pixel)
0.0	2.1/18.9	20 784/17 040	15.2	11.6/13.5	2.3
1.0	2.5/31.1	21 047/16 792	13.1	12.7/14.2	1.1
2.0	2.3/22.4	20 656/12 275	13.0	13.8/16.0	0.43
4.0	2.3/44.8	21 438/13 096	9.7	14.1/16.0	0.11
8.0	2.9/54.0	21 489/13 360	8.7	14.5/16.1	0.042

**TABLE IV.** Comparison of lossless compression algorithms in terms of total time to generate converted HDF5 file (reading raw gain + ADC frames, conversion to photon, frame summation, compression, and writing converted data to SSDs) processing time with XDS and compression factor for a large lysozyme dataset collected at the Swiss Light Source X06SA beamline JUNGFRAU 4M at 1.1 kHz using 12.4 keV x-rays, 100% beam transmission, and 0.088°/880 *μ*s steps. 2045 images (180° rotation) were taken for data analysis. For writing corresponding frequency is noted. Writing time was averaged over 10 runs while processing time over 20 runs due to smaller differences.

Compression algorithm	Writing time/frequency	Processing time	Compression (bit/pxl)
No compression	12.9 s/158 Hz	73.5 s	16.0
LZ4	6.4 s/320 Hz	73.3 s	6.8
Bitshuffle/LZ4	3.7 s/550 Hz	74.3 s	2.3
Zstd	6.3 s/324 Hz	75.4 s	2.8
Bitshuffle/Zstd	5.8 s/351 Hz	73.2 s	1.8
Gzip	66.7 s/31 Hz	75.1 s	2.4

To better investigate Bitshuffle performance, we have performed two experiments. First, presented in Table II, we have compared the compression ratio for different rounding schemes. For each 1 bit of higher precision, from rounding of 2 photons, there is roughly 0.9 increase in encoding per pixel. It clearly shows that compression only applies to low bits (encoding order of magnitude of pixels) and to encoding high precision. Therefore, increasing precision, especially beyond rounding to a single photon, will be expensive in space. In the second experiment, we have measured compression performance for original and already bit-shuffled data with different compression algorithms (compare Tables S1 and S3). While compression factors are better for bit shuffled data, as already seen, interestingly the same algorithm compresses bit shuffled data significantly faster. This is due to the fact that Bitshuffle data are more predictable and matches to the compression dictionary are made more often. However for non-shuffled data, the algorithm is less predictable. This effect is seen even at a CPU level, where LZ4 compression of not-shuffled data has a high number (25%) of CPU wrongly predicting loop outcomes (bad speculation), while there are close to none mispredictions in the case of bit shuffled data. The data also show that bit shuffling is a limiting step for combinations with both LZ4 and Zstd. In case conversion is performed on FPGA, the two steps can be decoupled. Bitshuffle would be most effective on FPGA, as these are bit order agnostic and such operation does not involve significant resources. FPGA implementation is considerably simpler than CPU, as Bitshuffle block fits into 20 lines of register transfer language or four lines of FPGA high-level synthesis C code, while CPU implementation requires more than 1000 lines of the C code. On the other hand, dictionary compression is difficult to perform on an FPGA chip, but effective on a CPU, so it should be optimally implemented there. With LZ4 performance well over 2 GB/s on a single core with bit shuffled data, less than 23 cores would be necessary to handle 46 GB/s of JUNGFRAU 10M, making implementation feasible.

Instead of fixed rounding, once could also apply a lossy compression algorithm to the floating-point outcome of conversion. A good choice would be one of the compression algorithms designed for scientific data that can guarantee certain precision. Here, we have evaluated the SZ algorithm^36^ with a relatively high error bound—while data quality is worse than in the case of rounding, a compression factor of 336× over raw data is impressive and allows us to pack a full dataset into a size similar to less than ten uncompressed images. One impact of the lossy compression is that the small local intensity fluctuation is flattened. This effect affects mostly background and has not much impact on strong reflections. However, it can average out weakest reflections into the background at high resolution. Therefore, the total number of accepted reflections after XDS CORRECT step is reduced at high resolution, as presented in Table III. The data completeness and multiplicity are reduced accordingly, which could result in a superficial reduction in R_meas_ at certain circumstances. The anomalous peak height, the precision of determining the last shell, and refinement statistics are all affected by lossy compression, but such a difference could be acceptable for some applications. Such compression could also be used in parallel with rounding schemes—an SZ compressed dataset could easily be taken home on a portable hard disk even for the most data = intensive serial crystallography experiments, for example for the ability to visualize images at user's home institution, while the higher precision data would remain available for processing in the high performance computing center at the synchrotron/XFEL facility.

Interestingly, a similar analysis was already performed by J. Holton in the past on diffraction images collected with charge-coupled device (CCD) detectors.^37^ In his work, a more elaborate scheme is explained, where spots should be compressed with a lossless algorithm, while the background is compressed with a lossy algorithm. He also pointed out that rounding or compression errors need to be always compared with other sources of errors—i.e., lossy schemes might not affect the outcome of the experiment at all if the magnitude of the uncertainty is small in comparison to e.g., counting statistics noise or the inability to correctly model observed Bragg spot amplitudes.^38^ Even very aggressive lossy compression schemes might be therefore good enough for high-throughput projects that do not require experimental phasing or do not rely on the quality of the highest resolution shell.

Another practical problem with pedestal subtraction is that some pixels, in the absence of photons, might sometimes get negative counts after conversion (e.g., −1) due to noise and the pedestal fluctuation combined effect. For serial crystallography, developed at XFEL sources with hybrid pixel integrating detectors, e.g., CSPAD,^31^ negative counts are not a new problem and are accepted by processing software, e.g., CrystFEL^32^ or cctbx.xfel^33^ as valid input. However, for modern rotational crystallography, where images form a time series and background can be estimated with higher precision than in serial method, negative observations are not well treated, since their occurrence is absent in photon counting detector data sets for which the analysis software is usually developed. For example, XDS^30^ is not accepting negative counts, as this would violate an assumption that photon counts follow the Poisson counting statistic. Here, temporary workarounds are to either fix negative numbers to zero or offset all pixel values by a given number, possibly explicitly taking each pixel pedestal variance into account. Therefore, for the time being, JUNGFRAU data generated for rotational and serial crystallography differ in the treatment of negative counts.^13^

## FILE FORMAT

To allow for seamless operation with MX processing programs, the converted images are stored in a container HDF5 format. For convenience, the file format used for experiments at Swiss Light Source was chosen to mimic data produced by the Dectris Eiger file writer interface.^39^ This allows the files to be read directly for visualization with Albula viewer and for processing with XDS through the Neggia plugin (Dectris) or with standard HDF5 plugins for CrystFEL.^40^ Further work is performed to adapt the software to produce HDF5 files fully compatible with NeXus NXmx data format,^41^ which also makes JUNGFRAU datasets directly readable by DIALS^42^ and cctbx.xfel.^43^ Full compatibility with the NXmx standard will necessitate a modification of the format itself. For example, the rounding mentioned in Pixel representation and compression section cannot be effectively described within existing NXmx metadata classes. The same applies to gain and pedestal maps used for the conversion process and description of the applied corrections (e.g., drift).

It should be noted that the current HDF5 library is not an ideal choice for high-performance applications. Due to historical reasons, it lacks thread safety, i.e., two threads operating within a single program cannot execute two HDF5 library calls in parallel, even if the scope of these calls is completely disjoint, and they access two independent HDF5 files. For this reason, hiding compression calls within HDF5 filter plugins should be avoided. Instead, data should be compressed before calling the HDF5 library and a direct chunk writer should be used. This is currently implemented for the Bitshuffle/LZ4, Bitshuffle/Zstd, LZ4, Zstd, and Gzip filters in our conversion routine, while SZ implementation is planned. While fully thread-safe HDF5 is not likely to improve performance, it will simplify the code and make it easier to implement newer algorithms into the code, thanks to the HDF5 plugin mechanism.

Another question is whether to save or not to save the raw unconverted data. It is important to keep such an option open in the design of the acquisition system for further research on JUNGFRAU behavior, as well as for troubleshooting. However, since knowledge of raw data is most likely not beneficial for MX data processing, and these data are poorly compressed, it is expected that incoming raw data from the detector will be converted on the fly and will not be stored, even on a short-term basis.

## PLANNED DATA ACQUISITION SYSTEM FOR JUNGFRAU 10M

Development schemes for IT products have recently changed. While for many years big progress was achieved in optimizing general-purpose products, mostly CPUs, this is no longer the case due to technical limitations of the manufacturing process.^44,45^ This has currently resulted in an increasing number of specialized processing units, which offer continuous growth in performance but require specialized programming techniques.^46^ These are, for example, already mentioned FPGAs and GPGPUs, but also specialized chips for machine learning (ML). All these are used currently for data science and artificial intelligence applications. Interestingly frameworks like Tensorflow, made for ML and utilizing GPGPU accelerators, can be also successfully applied to data analysis pipelines.^47^

Specifically, the results presented above show that the operation of a 46 GB/s JUNGFRAU 10 Mpixel will be challenging to run on mainstream-architecture server systems with software-only solutions. Instead, solutions that benefit from cutting-edge hardware mechanisms will be necessary to keep the solution simple and sustainable for any future increase in data rates. For example, real-time processing could be done on FPGAs, while x-ray image analysis performed on GPGPUs, as already shown for tomography data.^48^ Both devices have advantages over CPUs in terms of computing power and memory bandwidth, including 2^nd^ generation High Bandwidth Memory (HBM2) allowing for 450–900 GB/s memory bandwidth per single GPGPU^25^ or FPGA.^49^

While all these components can provide significant value to a data acquisition system, ensuring enough data bandwidth and seamless integration between them is crucial to successful implementation. Therefore, we have selected IBM POWER9 as a promising architecture to implement a data acquisition system for the most demanding detector. POWER architecture is strongly focusing on input/output performance, providing a bandwidth between components surpassing what is available with mainstream architecture. The three main interfaces are NVLink for CPU-GPGPU communication (up to 75 GB/s in each direction), OpenCAPI for CPU-FPGA communication (up to 25 GB/s in each direction), and PCIe 4.0 (up to 32 GB/s in each direction, double that of the PCIe 3.0).^50^ The benefit of using POWER interfaces, i.e., NVLink and OpenCAPI, is not only bandwidth, but these interfaces allow also for coherent memory access. FPGA board connected via OpenCAPI or GPGPU connected via NVLink sees host (CPU) virtual memory space exactly like the process running on the CPU, reducing the burden of writing reliable and secure applications. Memory coherency can be also available for PCIe FPGA accelerators installed in POWER9 servers via OpenCAPI predecessor, the Coherent Accelerator Processor Interface (CAPI). IBM also provides optimized software to benefit from the architecture, including the CAPI Storage, Network, and Analytics Programming (SNAP) framework^51,52^ that simplifies the integration of FPGA designs with POWER9, as well as optimized ML and data analysis routines for GPGPUs or FPGAs.^53^

**FIG. 4.**
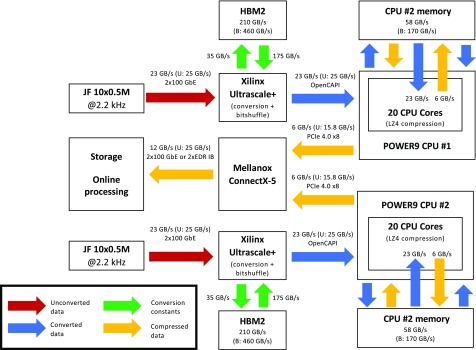
Conceptual design of data acquisition system for JUNGFRAU 10M for MX beamlines at the Paul Scherrer Institute with IBM AC 922 system, 2 Alpha-Data 9H3 FPGA boards and single 2 × 100 GbE Mellanox Connect-X network card. Detector operates at 2.2 kHz framerate. Maximal possible bandwidth of each interface is marked in parentheses according to hardware specifications^49,58^ (U: unidirectional bandwidth, B: bidirectional bandwidth). Assumes a compression factor of 4 with Bitshuffle/LZ4.

Current plans for the JUNGFRAU data acquisition system are presented in Fig. 4. These include a two-socket POWER9 server (e.g., AC922 or IC922) with two FPGA boards (e.g., Alpha Data 9H3 or 9H7) and Mellanox 2 × 100 GbE network card. In this design, FPGA boards accept incoming traffic of up to 46 GB/s. Packet sorting, conversion, and bit shuffling are all implemented in the FPGA. It will be also explored if spot finding or machine learning image analysis could be carried out on the fly on the FPGA to facilitate downstream data processing and/or avoid saving empty frames in serial crystallography experiments. Converted data, with additional metadata and annotations, are then transferred via a dedicated accelerator interface, OpenCAPI, to the host memory. When data are present in the CPU memory, they are compressed with the LZ4 algorithm on the CPU. Finally, compressed images are streamed directly to a data processing cluster via a queue protocol. Optionally, writing of HDF5 files and buffering on NVMe SSDs can be also done by a second server, to avoid file system overheads on the data acquisition system, with data being transferred between the two systems via RDMA. In this design, critical part of decoding is done on FPGA, which guarantees real-time processing of the data. Tasks of the CPU are limited to flow control and compression, which reduces competition to CPU memory access. Finally, lower energy consumption and heat production of FPGA, as compared for the same task on the CPU or GPU, allow one to handle higher throughput within a single computer box and reduce operation costs.^54^

## GENERAL OUTLOOK

Increasing data rates at next-generation synchrotron facilities and XFELs puts significant stress on computational and data storage infrastructure. This has consequences not only for the choice of computing technology but for science as well. Currently, a well-established paradigm in x-ray macromolecular crystallography is to save all the data at the highest possible precision. While in some cases this is indeed justified, such an approach is not sustainable in general. At some point, it will be only possible to operate above a certain frame rate if online data reduction is implemented by either a rounding scheme, lossy compression, or a veto mechanism for empty images in serial crystallography. Such choices should be made so that the imprecision and information loss in the data have a negligible impact on the outcome of the experiment. But the gain in the sustainable data rate will not only be beneficial for high-throughput applications but also could enable scientists to better explore what the next-generation light sources can offer.

## METHODS

Data acquisition and all the calculations were performed on an HPE DL580 Gen10 server. The server was equipped with 4 Intel Xeon Gold 6146 CPUs (3.2 GHz; 12 core/CPU), 1.5 TB RAM (DDR4–2666), four 3.2 TB PCIe Non-Volatile Memory Express (NVMe) SSDs, 100 GbE Mellanox Connect-X 4 network card, and four 2-port 10 GbE Mellanox Connect-X 4 Lx network cards. For mid-term storage of images, an external array of 24 disks, each 12 TB, was attached via a Serial Attached SCSI (SAS) controller and operated with a Zettabyte File System (ZFS), allowing roughly 1.5 GB/s writing speed. The server was capable of handling 9 GB/s JUNGFRAU 4M at 1.1 kHz, but only in a mode, where incoming raw gain+ADU data were first saved to the RAM disk and later converted, compressed, and written to external storage.

The system was running Red Hat Enterprise Linux v. 7.6. Receiving of data and communication with the detector were handled by SLS Detector package v. 3.1.4. Software development for the converter was performed with Intel Parallel Studio XE v. 2018 and v. 2019, including the Intel C/C++ compiler. Performance analysis was performed with Intel Advisor and Intel VTune Amplifier. Vectorization of the code was achieved by enforcing array memory alignment and *ivdep* pragmas, no intrinsic libraries were used. Optimized LZ4 and gzip routines were used from the Intel Performance Primitives toolset. Zstandard compression algorithm v. 1.4.4 was used. The Bitshuffle filter was modified to accommodate Zstandard compression and is available to download from the authors' Github. The block size for Bitshuffle/Zstandard was increased to 64 kB = 32678 × 16-bit, with significantly better compression performance. For Bitshuffle/LZ4, the gain in the compressed size was negligible and the default block size of 8 kB = 4096 × 16-bit was used. SZ compression v. 2.1.7 was used. High-Level Synthesis code for FPGAs was developed and synthesized with Xilinx Vivado HLS v. 2019.2 for Xilinx Ultrascale+ XCVU33P-2E FPGA, as installed on the Alpha Data 9H3 board.

A lysozyme test crystal was prepared in the same way as before.^13^ Measurement was carried out at the X06SA beamline. The JUNGFRAU 4M detector was used, composed of 8 modules each having 1024 × 512 pixels. The detector was cooled to −12 °C to achieve a long integration time of 840 *μ*s, with a frame time of 880 *μ*s (1.1 kHz). X-ray energy was set to 12.4 keV and no beam attenuation was used. The crystal was measured with 100°/s rotation speed, resulting in 0.088° per single image, as before.^13^ Data collection started roughly 3343 frames (2.9 s) before the sample was illuminated with x-rays. To account for shutter opening time, frames starting with number 3500 were converted into photon counts, while previous frames were used to calculate and track the G0 pedestal. Data were processed with XDS with parallelization set to 4 jobs, each over 12 CPUs.^55^ The R_meas_^31^ data quality indicator was extracted from the XDS output. The anomalous peak height was calculated with ANODE^56^ based on the 6G8A lysozyme model deposited previously by us to the PDB.^13^ Refinement was performed with phenix.refine^57^ using the same input file, as previously, optimized for high resolution structure. The same free reflection set was used for all refinement runs of the same system.

## CODE AVAILABILITY

Conversion code is available at https://github.com/fleon-psi/JFConverter. The modified Bitshuffle filter to accommodate Zstandard is available at https://github.com/fleon-psi/bitshuffle. Lysozyme unconverted JUNGFRAU 4M images (∼150 GB) are available from the PSI Public Repository (https://doi.org/10.16907/808de0df-a9d3-4698-8e9f-d6e091516650).

## SUPPLEMENTARY MATERIAL

See the supplementary material for additional results on lossless compression throughput (Tables S1–S3).
